# Population Structure Analysis and Association Mapping for Turcicum Leaf Blight Resistance in Tropical Maize Using SSR Markers

**DOI:** 10.3390/genes13040618

**Published:** 2022-03-29

**Authors:** Bhupender Kumar, Mukesh Choudhary, Pardeep Kumar, Krishan Kumar, Sonu Kumar, Brijesh Kumar Singh, Chayanika Lahkar, Meenakshi, Pushpendra Kumar, Zahoor Ahmed Dar, Rakesh Devlash, Karambir Singh Hooda, Satish Kumar Guleria, Sujay Rakshit

**Affiliations:** 1 ICAR-Indian Institute of Maize Research, Ludhiana 141004, India; bhupender.icar@gmail.com (B.K.); mukesh.agri08@gmail.com (M.C.); pardeep.kumar656@gmail.com (P.K.); krishjiwra@gmail.com (K.K.); nagarsonu72@gmail.com (S.K.); brijeshsingh714@gmail.com (B.K.S.); chayanika.iimr@gmail.com (C.L.); sharmameenakshi986@gmail.com (M.); pushpendrakum222@gmail.com (P.K.); hoodaks@gmail.com (K.S.H.); 2 Dryland Agriculture Research Station, SKUAST-K, Srinagar 190001, India; zahoorpbg@gmail.com; 3 CSKHPKV, HAREC, Bajaura 175125, India; rdevlash@yahoo.in (R.D.); skg0612@rediffmail.com (S.K.G.); 4 ICAR-National Bureau of Plant Genetic Resources, New Delhi 110012, India

**Keywords:** maize, turcicum leaf blight, simple sequence repeats, genetic diversity, population structure, general linear and mixed linear models, genome-wide association study

## Abstract

Maize is an important cereal crop in the world for feed, food, fodder, and raw materials of industries. Turcicum leaf blight (TLB) is a major foliar disease that can cause more than 50% yield losses in maize. Considering this, the molecular diversity, population structure, and genome-wide association study (GWAS) for TLB resistance were studied in 288 diverse inbred lines genotyped using 89 polymorphic simple sequence repeats (SSR) markers. These lines werescreened for TLB disease at two hot-spot locations under artificially inoculated conditions. The average percent disease incidence (PDI) calculated for each genotype ranged from 17 (UMI 1201) to 78% (IML 12-22) with an overall mean of 40%. The numbers of alleles detected at a locus ranged from twoto nine, with a total of 388 alleles. The polymorphic information content (PIC) of each marker ranged between 0.04 and 0.86. Out of 89 markers, 47 markers were highly polymorphic (PIC ≥ 0.60). This indicated that the SSR markers used were very informative and suitable for genetic diversity, population structure, and marker-trait association studies.The overall observed homozygosity for highly polymorphic markers was 0.98, which indicated that lines used were genetically pure. Neighbor-joining clustering, factorial analysis, and population structure studies clustered the 288 lines into 3–5 groups. The patterns of grouping were in agreement with the origin and pedigree records of the genotypesto a greater extent.A total of 94.10% lines were successfully assigned to one or another group at a membership probability of ≥0.60. An analysis of molecular variance (AMOVA) revealed highly significant differences among populations and within individuals. Linkage disequilibrium for r^2^ and D′ between loci ranged from 0 to 0.77 and 0 to 1, respectively. A marker trait association analysis carried out using a general linear model (GLM) and mixed linear model (MLM), identified 15 SSRs markers significantly associated with TLB resistance.These 15 markers were located on almost all chromosomes (Chr) except 7, 8, and 9. The phenotypic variation explained by these loci ranged from 6% (umc1367) to 26% (nc130, phi085). Maximum 7 associated markers were located together on Chr 2 and 5. The selected regions identified on Chr 2 and 5 corroborated the previous studies carried out in the Indian maize germplasm. Further, 11 candidate genes were identified to be associated with significant markers. The identified sources for TLB resistance and associated markers may be utilized in molecular breeding for the development of suitable genotypes.

## 1. Introduction

Maize (*Zea mays* L., 2n = 20) is considered an important feed, fodder, and staple food throughout the world and is popularly used as a model organism in plants due to its high genetically diverse nature [1,2,3]. Increasing population, climate change, and productivity constraints have enhanced the demand forpoultry feed, fabric starch production, pharmaceutical, cosmetic industry, high-quality corn oil, protein, alcoholic quencher, and biofuels [4,5]. Therefore, the need of the hour is to improve maize for various economical traits. Maize is a highly out-crossed crop with enormous genetic diversity that confers a significant level of heterosis for hybrid development. The availability of adequate genetic diversity is the strength of any crop-breeding program. Furthermore, the use of diverse types of genetic materials such aslandraces and wild species as donors is highly recommended so as to enrich the existing germplasm with favorable alleles [6,7].

Worldwide, only 10% of the total available genetic diversity in maize has been used so far in breeding programs. On the other hand, the development of modern varieties, replacement of old landraces, increased population and climate change are the major factors of reduction in allelic diversity (genetic erosion) in maize [1,8]. Therefore, ananalysis of genetic diversity and population structure of different germplasm is very useful for broadening the genetic base and selecting the promising parental combinations for hybrids development [3,9,10,11]. Furthermore, the information on population structure and genetic diversity are useful to study the markers traits association for different economical traits.

Different approaches are available for the analysis of genetic diversity such asmolecular, biochemical, and phenological approaches. In the present era, DNA-based markers (molecular markers) are frequently used for genetic diversity and grouping of the populations [12]. Among the various high throughput DNA-based marker techniques available, single nucleotide polymorphisms (SNPs) and simple sequence repeats (SSRs) are the markers of choice because they are co-dominant in nature, locus-specific, reproducible, highly informative, and easy to use [13]. These markers are not influenced by environmental changes and are thereforeuseful in genetic diversity, population structure, and mapping studies [12,14]. Moreover, SSR markers are more informative than biallelic SNP markers because they can detect multiple alleles per locus [15]. Van Inghelandt et al. [9] reported SSRs to be 7 to 11 times more accurate than SNPs. Moreover, SSR markers have been successfully and efficiently used to assess the extent of genetic diversity and population structure in maize [3,16,17,18,19,20,21,22,23].

For a sustainable genetic gain in maize, the development and deployment of productive hybrids from diverse lines that performbetter under biotic and abiotic conditions is very much required [24]. Amongst the biotic conditions, turcicum leaf blight (TLB), also known as northern corn leaf blight (NCLB) caused by *Exserohilumturcicum* (Pass) Leonard and Suggs (Teliomorph = *Setosphaeria turcica* (Luttrell) is an important foliar disease prevalent worldwide and reported to cause up to 50% yield losses in maize [25]. Generally, it is more severe in regions where moderate temperatures and high humidity prevail [26]. TLB resistance is complex and polygenic in nature [27,28]. In India, TLB is a more common and severe disease of maize prevalent in almost all maize growing ecologies of the country [26]; therefore, there is an urgent need to breed for its resistance. In conventional breeding, genotypes are selected/rejected based on phenotypic expression, which is governed by many environmental factors. Furthermore, it is more time-consuming and innovative approaches need to be explored for its resistance breeding.Different approaches are available to identify genomic regions viz., conventional linkage-based mapping, and genome-wide association mapping [29]. The efficiency of conventional linkage mapping depends upon genetic background, size of the population, diversity between two parents and the number of loci used. Generally, linkage mapping has a lowerresolution compared to the GWAS, because of the limited number of recombination events [30]. Nevertheless, this approach has been extensively used in maize and other plant species, even before past two decades, for the mapping of the gene (s) [25,31]. In the last decade, anincreasing use of GWAS has been noted to identify genomic regions for various useful breeding traits in maize and other crops [32,33,34]. GWAS explores the historical and evolutionary recombination events at the population level [34]. Additionally, the diverse panel used in the studies provides opportunities toidentify multiple alleles for a trait as well [29,30]. The population structure and genetic relatedness may lead to a spurious association in GWAS. However, analyzing the genotypes for their structure and kinship relation using suitable tools, e.g., STRUCTURE and Tassel 3, respectively, can help in eliminating false association between a marker and target trait [35].

At the globallevel, several reports on the genetic characterization of maize germplasm and genomic regions for TLB resistance are available [36,37]. Most of these studies are based on temperate to sub-temperate maize germplasm and used a conventional linkage-based mapping approach for marker-trait linkage identification. With regard toIndian maize germplasm, only one report is available so far, in whichthe authorsused conventional linkage-based mapping in an F_2:3_ mapping population to identify genomic regions for TLB resistance [25]. Similarly, Rashid et al. [23] evaluated International Maize and Wheat Improvement Center (CIMMYT) panel in India for TLB disease and identified the loci associated with TLB resistance using GWAS. Germplasm development and its characterization is a continuous process. Many new lines have been introduced in the breeding programme. Therefore, anassessment of their genetic diversity and population structure will contribute toefficient utilization in the ongoing breeding programme. Furthermore, in India, limited efforts have been made toidentify genomic regions for TLB resistance in maize. Considering the importance of genetic diversity, population structure analysis, and TLB resistance in maize, the current study was planned with the objectives of the genetic characterization of existing and the development of a large set of diverse maize inbred lines (originated from CIMMYT and nine different maize research centers in India) with SSR markers and the identification ofgenomic regions using the GWAS approach for TLB resistance in tropical maize.

## 2. Materials and Methods

### 2.1. Plant Materials and DNA Isolation

In this study, a set of 288 genetically diverse maize inbred lines (Table S1) was used for the assessment of genetic diversity, population structure and marker-trait association for TLB resistance in tropical maize. These lines originated from ten different centers/organizations and derived from diverse source populations. Out of 288, 75 lines were obtained from CIMMYT and the remaining 213 were from nine different maize research centers working in India. Furthermore, a total of 212 lines among these were f the normal field corn group, 75 were from quality protein maize (QPM) and one was from the popcorn group. Data were collected from randomly selected plants for TLB disease, days to anthesis (DTA), plant height (PHT; cm) and ear height placement (EHT; cm). DNA was extracted from the bulked leaf tissues (15 days old seedling) of five randomly selected plants of each genotype grown in the glasshouse. Each leaf sample was grounded with liquid nitrogen using the CTAB extraction method with slight modifications. The total DNA quantity and quality were estimated using 1% Agarose gel in electrophoresis with uncut lambda DNA as standard. The quantified DNA samples were diluted to a concentration of 50 ng/μL for use in polymerase chain reactions.

### 2.2. Disease Screening

During the year 2018–2019, a diverse set of 288 maize inbred lines from 10 different maize research centers/organizations were characterized for DTA, PHT and EHT at the Delhi location (Table S1). They were subjected to field screening for turcicum leaf blight resistance under artificial inoculated conditions at two hot-spot locations, viz., Bajaura and Srinagar with anaugmented design. The inoculum of TLB was multiplied on whole sorghum grains and each line was infested by placingit in the whorl of 35-day-old plants. Inoculation was repeated after one week of the first inoculation. The disease screening was undertaken by maintaining a conducive environment during the whole cropping season. For the effective spread of the pathogen, water was sprayed in plant whorls using a knapsack sprayer at 3 days intervals in case of no rain. A disease rating was performedafter the grain-filling stage using a 1.0 (resistant)–9.0 (susceptible) scale and was utilized to estimate the percentage of disease incidence (PDI) as per Hooda et al. [26].

### 2.3. Genotyping Using SSR

A set of 140 pairs of SSR primers uniformly distributed throughout the maize genome were initially tested in a representative set of 288 inbred lines (total 12), of which 89 were found to be polymorphic. The PCR reactions were performed as per the standard protocol [38,39]. The amplified DNA samples with a 50 bp ladder were separated by electrophoresis in a 3% metaphor gel. The allele’s size in terms of base pairs was determined based on their relative positions in the gel. The details of SSRs regarding their primer sequences, chromosome position, annealing temperature and polymorphic information content (PIC), are provided in Table S2. The genotyping data of 89 polymorphic SSR markers were used for genetic diversity, population structure, and marker-trait association analysis for disease resistance.

### 2.4. Genetic Diversity and Population Structure Analysis

The different genetic diversity parameters such as alleles per locus, polymorphic information content (PIC), marker index (MI), the diversity index (DI), and heterozygosity were calculated using PowerMarkerV.3.25 [40]. These parameters help to understand the frequency of an allele, heterozygous loci, and the quantum of polymorphism [41] in the selected set of genotypes. POPGENE software version v.1.32 [42] and Excel were used to calculate different genetic parameters such asthe number of observed (Na) and effective allele (Ne) numbers at a locus [43]. The expected homozygosity and heterozygosity were calculated using Nei’s [44] gene diversity statistics. The neighbor-joining tree, on the basis of distance matrix, and a factorial analysis were performed using DARwin software 6.0.21 and iTOL [45,46,47]. The analysis for molecular variance (AMOVA) between the populations and within them (testing F_st_ by 9999 random permutations) was performed usingGenAlEx version 6.5 software [48]. The population structure was studied using STRUCTURE v 2.3.3 software for the assessment of sub-populations and genetic relationships among the 288 genotypes [49]. The project was run with the set parameters of the population admixture model and the allele frequency correlated.The hypothetical sub-populations in the panel were considered as K = 1 to 10 with three independent runs for each K.The length of the burn-in period and the number of iterations was set at 1, 50,000. The optimum value for K was wherever the subsequent values of ln Pr (*X*|*K*) stopped varying significantly [49]. The pedigree record and breeding history of the lines were also considered while deciding optimum sup-populations. Inbred lines with membership probability values of ≥0.60 were assigned to the same group, while those with <0.60 probability were treated as mixed [50].

### 2.5. Linkage Disequilibrium and Marker-Trait Association Analysis

Linkage disequilibrium (LD) values for r^2^ [51] and D′ [52] between SSR loci on chromosomes were calculated using Tassel 3.0 (https://tassel.bitbucket.io/) following permutation test of 10,000. A kinship matrix (K) and marker-trait association analysis were done in Tassel 3.0 using the genotypic data of 89 polymorphic markers and phenotypic data on PDI of TLB disease for a set of 288 diverse lines. The association study was conductedusing a general linear model (GLM) with Q matrix (individuals probability of membership in the population) [53] as well as a mixed linear model (MLM) with kinship (K) and Q matrix [35]. Finally, the associated markers were filtered out based on theR^2^ of the marker at a very high significance (*p* < 0.001) level and with the lowest false discovery rate(FDR). These markers were physically located on chromosomes using the MaizeGDB database, as well asnucleotide and primer blast tools. Furthermore, the putative candidate gene belonging to the selected markers was retrieved from MaizeGDB.

## 3. Results

### 3.1. Phenotypic Variability

**Figure 1:**
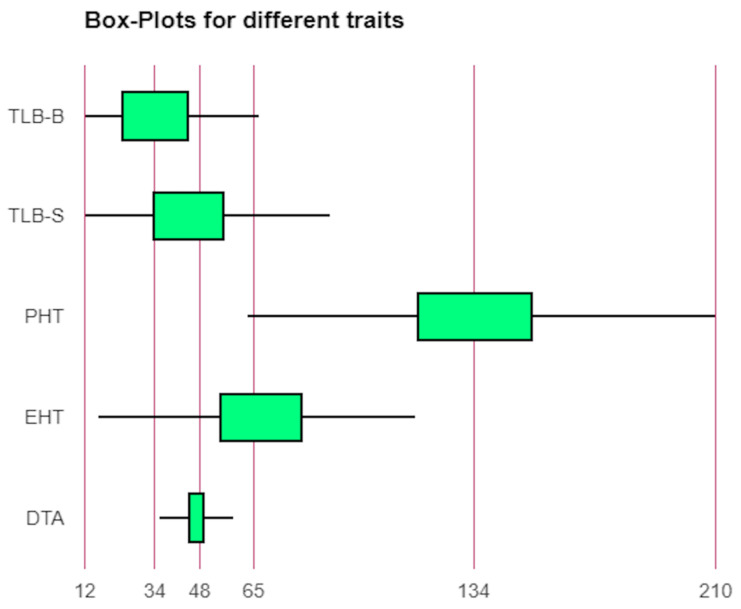
Phenotypic variability for days to anthesis (DTA), ear height placement (EHT), plant height (PHT), and turcicum leaf blight (TLB) at hots-spots location Srinagar (TLB-S) and Bajaura (TLB-B).

Sufficient variability was observed for TLB disease, DTA, PHT, and EHT placement (Table S1, Figure 1 and Figure S1). The average PDI of TLB ranged from 17 (UMI 1201) to 78% (IML 12-22) with an overall mean of 40% (Table S1). All types of responses to the TLB, viz., resistant, moderately resistant, moderately susceptible, and susceptible were observed in the panel. Some of the genotypes, namely UMI 1201, BML 7, DML 310, CML 542W, IML 12-116, P72c1 × brasil1177-2, DQL 1017-2, DQL 779-1 were found to beresistant to moderately resistantagainst TLB acrossthe locations. Similarly, the PHT and EHT placement ranged from 63 (DQL 653-3-1) to 210 cm (UMI 1200) and 16 (IML 15-65) to 116 cm (CM 207), with a mean of 135.3 cm, and 67.52 cm, respectively.

### 3.2. Genetic Diversity and Population Structure Analysis

Out of the 140 SSR markers used for initial screening, 89 (63.6%) were polymorphic in 288 genotypes, with a total of 388 alleles. The number of alleles detected at a locus ranged from twoto nine. Markers *umc2303*, *phi085* and *umc 2284* exhibited the maximum number of alleles (9), and a group of markers, viz., *bnlg1458*, *bnlg2086*, *bnlg421*, *phi038*, *phi108411*, *bnlg128*, *phi059*, *umc1367*, *umc1196*, *umc1607*, *umc1161*, *umc1913*, *umc2324* and *umc1296* recorded the minimum number of alleles (2 alleles) in the genome. Similarly, the effective number of alleles (ne) ranged from 2.58 (*umc2077*) alleles to 7.73 (*umc2303*) per locus (Table 1 and Table S2). The details of 89 polymorphic markers are provided in Supplementary Table S2. The PIC of each marker ranged between 0.04 (*umc1161*, *umc1296*) and 0.86 (*umc2284*) (Table S2). Out of 89 markers, 47 markers were highly polymorphic (PIC ≥ 0.60) (Table 1).

The marker index (MI) ranged from 3.15 (*umc1161*) to 370.80 (*phi085*) with an average value of 107.24 (Table S2). Similarly, the average value of the diversity index (DI) was 0.86, and ranged between 0.52 (*umc1161* and *umc1296*) to 0.99 (*phi085*). The marker attributes, viz., PIC, MI, and DI are routinely used to evaluate the informativeness of the primers. In the current study, a PIC value ≥ 0.60 was observed in over 50% of the markers. This indicated that the SSR markers used were very informative and can be useful in the assessment of genetic diversity, population structure, and marker–trait association studies. In the current study, the SSR primer set, *phi085*, appeared to be highly informative, having high PIC, MI, DI, and Na (Table 1 and Table S2). The value of the observed homozygosity for highly polymorphic markers ranged from 0.73 to 1.00 with an overall average of 0.98 (Table 1).

**Figure 2:**
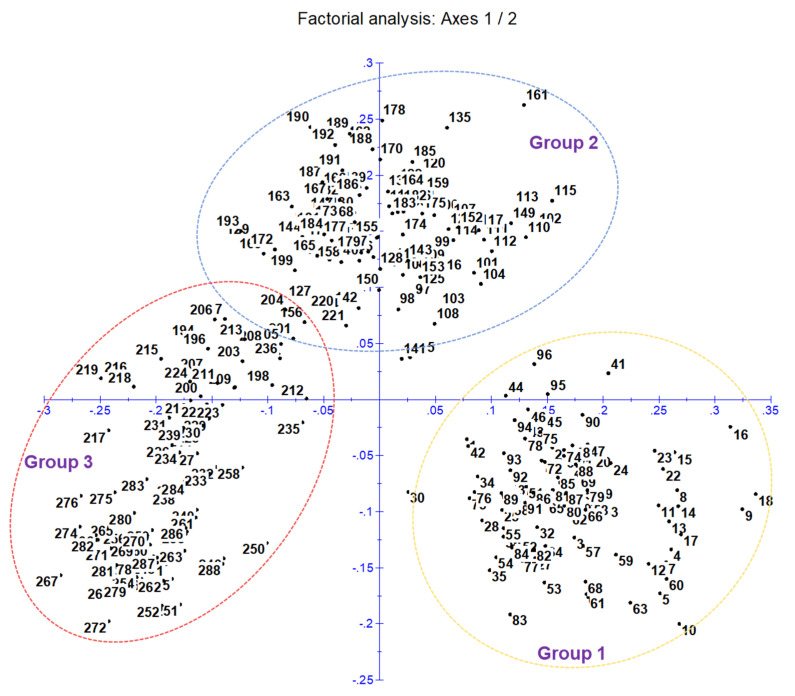
Principal coordinate analysis (factorial analysis) classifies 288 genotypes into three main clusters and agrees with neighbor-joining clustering.

**Figure 3:**
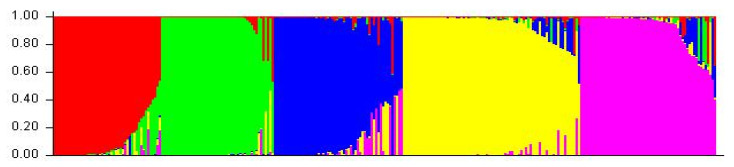
Population structure of 288 maize inbred lines revealed by 89 polymorphic SSRs markers at K = 5. Each inbred line is represented by a vertical line which indicates the membership coefficients for each individual. The five groups are shown in different colors: G1 (Green), G2 (Red), G3 (Purple), G4 (Yellow), and G5 (Blue). See Table S1 for details on group membership.

A cluster analysis based on the unweighted neighbor-joining method grouped 288 inbred lines into three main clusters (Figure S2). Furthermore, the main clusters-1 (yellow), 2 (blue), and 3 (red) were divided into two sub-clusters, each representing 33.34%, 32.64%, and 34.02% of the total 288 inbred lines, respectively. Similarly, the factorial analysis also revealed three major groups, as observed in clustering (Figure 2). The five optimal sub-populations were identifiedin the structure analysis (Figure 3). Inbreds with a membership probability of ≥0.60 were assigned to the same group and if they had a membership probability of less than this value, they was considered as mixed (not assigned to any of the five groups). Of the 288 inbred lines, 271 (94.10%) were assigned into either one of the five groups and the remaining 17 lines (5.90%) were categorized as mixed (Table S1). The grouping behavior of the lines was mostly observed as per their center from where they originated and in accordance with the pedigree record (Table S1).

AMOVA is a suitable criterion by which to assess the overall distribution of diversity within and among populations. AMOVA revealed highly significant differences among populations and within individuals. Approximately 93% (88% of the total variance among individuals and 5% within individuals) of the variation was within sub-populations, while only a 7% variation was found among populations (Table 2).

The AMOVA results revealeda higher level of genetic variation among the individuals in groups than between different groups of populations. Wright’s F statistics (F_is_, F_it_, F_st_) was calculated to observe the molecular variation within and across the individuals of the population. The F_is_ (within individuals across the whole population) and F_it_ (among individuals within a population) values were observed as 0.94 and 0.95, respectively. The F_st_ (fixation index; between sub-populations or groups of populations) for the polymorphic loci across all accessions was calculated as 0.07. The value of F_st_ ranged from 0 to 1, with 0 indicating complete panmixis (two populations are interbreeding freely), whereas 1 implies that two populations do not share any genetic diversity. The result of F_st_ indicated a low to medium differentiation between subgroups of the population [54].

### 3.3. Marker Trait Association Analysis

**Figure 4:**
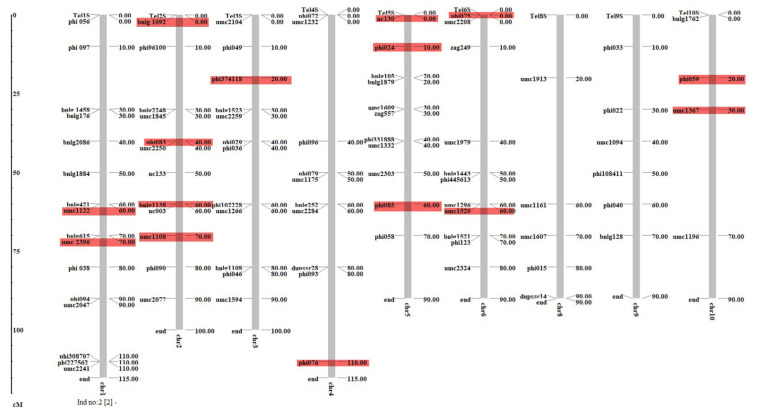
Chromosomal localization of polymorphic SSRs used for genotyping of 288 diverse lines. No polymorphic markers were found on Chr 7. The marker highlighted in red colors were found associated with TLB resistance. The details of all these markers have been given in Table 3 and Table S1.

LD can be defined as the non-random association between different loci on either the same or on different chromosomes. The r^2^ and D′ values indicate the existence of significant LD between markers on the same or on different chromosomes. The value for r^2^ between the marker pairs ranged from 0.00 to 0.77; however, the values for D′ ranged from 0 to 1.0. A marker trait association analysis was performed using GLM (Q) and MLM (Q + K) implemented in Tassel 3.0. A total of 15 SSR markers, viz., one on chromosomes (Chr) 3 and 4, two on Chr 1, 6, and 10, three on Chr 5 and four on Chr 2 were found to be significantly (*p* < 0.001, with FDR 0.0001 to 0.04) associated with TLB resistance in GLM (Figure 4, Table 3). The phenotypic variation explained by these loci ranged from 6% (*umc1367*) to 26% (*nc130*, *phi085*). None of the markers were found to be significantly associated with TLB in MLM at low FDR. Furthermore, a total of eleven putative candidate genes were identified to be associated with significant markers.

The putative candidate genes belonging to selected markers are as follows: *Zm00001eb253820*: glutamine synthetase (phi085); *Zm00001eb210620*: *LOC100193664* (nc130); *Zm00001eb212940*: opaque2 heterodimerizing protein 2 (phi024); *Zea mays* metallothionein-like protein type 2: *LOC100283295* (phi374118); *Zm00001eb260140*: ferredoxin I (Fd) isoprotein (phi075); *Zea mays* catalase (Cat3): *L05934.1* (phi076); *Zm00001eb038580* (umc1122); *Zm00001eb080380*: prp2—pathogenesis-related protein2 (phi083); *Zm00001eb292690*: tcptf23—TCP-transcription factor 23 (umc1520); *Zm00001eb407710*: aasr1—abscisic acid stress ripening1 (phi059) and *Zm00001eb410600* (umc1367).

## 4. Discussion

### 4.1. Genetic Diversity and Population Structure

Genetic diversity and population structure analysis are important tools for germplasm characterization and subsequent utilization in traits improvement. Apopulation with a high level of genetic diversity helps to broaden the genetic base in any breeding program. To assess the genetic diversity in maize genotypes, SSR markers remain a marker of choice due to their co-dominant and multi-allelic nature, abundance, and the specificity of the locus [9]. In this study, a distance-based clustering approach using 89 polymorphic SSRs was used to evaluate the genetic diversity and population structure among the 288 inbred lines. A total of 388 alleles with a range of twoto nineper locus in the present study, indicate the wide range of diversity among the genotypes [19,20,21,22,23,55]. Lanes et al. [19] and Vega-Alvarez et al. [55] reported 471 and 649 alleles, respectively, which were higher in comparison to the present study (388 alleles), while Xiao et al. [56] reported a relatively lower number of alleles, i.e., 145. Similarly, an average of 9.60 (range of 4 to17), 2.96 (2 to 4) and 14.57 alleles per locus were reported in maize using SSRs [3,9,38], respectively. The differences in the number of alleles across studies may be due to the use of different genetic materials and the number of markers. The average PIC value of 0.54 (range from 0.04 to 0.86) indicates the presence of more informative allelic variations in the panel [41]. The high polymorphic markers could be used effectively for fingerprinting and QTL mapping studies. The high values of homozygosity observed in the current study indicate that the inbred lines are genetically pure and therefore can be used in hybrid breeding [3]. Since maize is a highly cross-pollinated crop, some amount of residual heterozygosity persists.

Neighbor-joining clustering grouped the accessions into three major clusters. Clear patterns of grouping were observed as per the center that developed the lines and in accordance with the pedigree/source populations. Furthermore, cluster-1 mainly accommodated the lines from IIMR (87), with a few lines (9) from five AICRP centers and research partners working in India. Conversely, cluster-2 carries most of the lines of CIMMYT (69) and the remaining (25) from four different AICRP partners. The sister lines sharing similar or related pedigree/source populations were generally grouped into one cluster. The majority of lines of AICRP Karnal, which are derived from CIMMYT materials, were grouped into cluster-2, largely accommodating the CIMMYT lines. Cluster-3 included the maximum lines of IIMR (84) and the remaining 14 from almost all maize research partners. The model-based analysis using STRUCTURE identified five genetically differentiated groups among all lines. In previous studies, Aci et al. [21] used 47 maize landraces and reported two sub-populations, whereas Sofi et al. [23] performed anadmixture model-based approach in 25 maize accessions and reported seven groups. Despite the slight variation in the number of groups and clusters in Neighbor-Joining clustering, factorial analysis, and population structure, a similar pattern of grouping was observed in the current study. Adu et al. [3] also observed a different number of groups based on clustering (fiveclusters) and population structure analysis (twosub-populations) in maize. Considering the membership probability of ≥0.60, 17 inbred lines were not assigned to any of the groups and were therefore marked as mixed (Table S1). The remaining 271 lines were assigned to either of the groups. This shows that the lines included in the study are highly homozygous and contained a genetically distinct group [50]. These inbred lines can be a good component of an association mapping panel for GWAS studies. A low to moderate level of genetic differentiation (F_st_ = 0.07), confirmed by a low rate of inbreeding, shows a high genetic identity level of the populations under study. Genetic differentiation (F_st_ = 0.07) among our populations can be ranked bythose found in the American Southern accessions (0.12) [57] andSahara accessions [22].

### 4.2. Markers Trait Association Analysis

Turcicum leaf blight is a serious disease of maize (59). The occurrence of disease in the field depends upon many field and climatic factors, thereforescreening should be carried out in hot-spot sites under artificial inoculated conditions. Although several practices are available for TLB management in maize, the identification and exploration of resistance sources are more sustainable and effective [58]. Sufficient variability was observed in the panel for TLB disease. The disease-resistant sources identified can be utilized for introgression into tropical maize. Furthermore, highly susceptible and tolerant lines can be utilized to map populations for genetic/genomic studies in maize [39,59].

Finding genomic regions and markers associated with TLB resistance can effectively help with the issues of field screening. Association analysis provides ample opportunities to dissect complex traits using natural variation in the germplasm [29]. However, afalse association is the major constraint in this approach that needs to be tackled beforehand [29,34,60]. The maximum number of SSRs loci were found to be associated with TLB resistance forchromosomes 2 and 5. No significant association was found forchromosomes 8, and 9, (Table 3, Figure 4). The SSR primer set, *phi085* located on Chr 5, appeared to be highly informative withhigh PIC, MI, DI, and Na, and was also found to be significantly associated with TLB resistance. Generally, with regard toIndian maize germplasm, only a few reports are available on mapping genomic regions for TLB resistance. However, recently, Ranganatha et al. [25] mapped the QTLs for TLB resistance using an F_2:3_ mapping population derived from a cross between CML 153 (susceptible) and SKV 50 (resistant). Out of the three significant QTLs identified, one was mapped on Chr 2 (2.06) and 5 (5.04–5.05). In the current study, we also identifiedseven markers, such as the four located on Chr 2 (*bnlg1092* (2.00), *phi083* (2.04), *bnlg1138* (2.06), *umc1108* (2.07)) and three on Chr5 (*nc130* (5.00), *phi024* (5.01), *phi085* (5.06)) (Table 3, Figure 4) and explained phenotypic variation from 12 to 26%. We found some of the markers at exactly the same bin location as mapped by Ranganatha et al. [25]. Similarly, the genomic regions were also mapped on Chr 1, 2, 5, 8, and 9 by Xia et al. [36] using recombinant inbred lines as a mapping population. These regions may be considered important for understanding the molecular basis and molecular breeding for TLB resistance in tropical maize. Rashid et al. [23] also identified SNPs for TLB on Chr 1, 7, 8 and 10 using GWAS. The putative candidate genes and new regions identified here may further be explored for validation and synthesis of gene-based markers for TLB resistance.The markers found to be associated with TLB resistance in this study would be useful for molecular breeding and further fine mapping TLB resistance, with the subsequent addition of markers.

## 5. Conclusions

This study attempted to understand the genetic diversity and population structure of 288 maize inbred lines that originatedfrom diverse sources and breeding programs in India. Moreover, using GWAS, the molecular markers associated with TLB disease resistance in tropical maize were identified. Sufficient genetic variation was reported for morphological traits, TLB disease response, and molecular markers in 288 inbred lines. The presence of more alleles per locus and high marker polymorphism indicated the existence of a broad genetic base in the germplasm. The high homozygosity in the panel indicated the purity of inbred lines. Based on the various statistical methods, the grouping of germplasm into different groups agreed to a greater extent with the origin and pedigree records of genotypes. A total 94.10% lines were successfully assigned to one or another group at a membership probability of ≥0.60. In GWAS, 15 markers were found to be significantly associated with TLB disease resistance in tropical maize. Genomic regions were identified on almost all chromosomes except 8, 7 and 9. The selected regions identified on Chr 2 and 5 mostly matched with the previous mapping studies conducted using Indian maize germplasm and exotic germplasm. The identified resistance sources, markers associated with TLB resistance and candidate genes may be validated and utilized in molecular breeding for the development of suitable genotypes.

## Figures and Tables

**Table 1:** Genetic characteristic of 47 highly polymorphic SSR loci (>0.60) across the 288 maize genotypes.

S. No.	SSR Locus	Bin Loc	PIC	MI	DI	Na	Ne	Obs_Hom
1	*phi 056*	1.00	0.76	180.56	0.96	6	4.21	1.00
2	*bnlg 1884*	1.05	0.67	133.08	0.93	5	3.42	1.00
3	*umc 1122*	1.06	0.76	179.75	0.96	6	6.20	1.00
4	*bnlg615*	1.07	0.61	97.17	0.90	4	2.62	0.92
5	*umc 2396*	1.07	0.73	143.61	0.95	5	4.79	1.00
6	*umc 2047*	1.09	0.72	113.31	0.93	4	3.55	1.00
7	*phi308707*	1.10	0.67	105.53	0.92	4	3.01	1.00
8	*phi227562*	1.11	0.64	125.79	0.93	5	2.77	1.00
9	*bnlg 1092*	2.00	0.68	135.24	0.94	5	3.19	1.00
10	*phi 96100*	2.01	0.63	124.03	0.93	5	2.73	0.97
11	*bnlg 2248*	2.03	0.74	145.71	0.95	5	4.26	1.00
12	*umc1845*	2.03	0.66	130.65	0.93	5	3.01	0.98
13	*phi 083*	2.04	0.71	139.48	0.94	5	3.47	1.00
14	*nc 133*	2.05	0.71	169.46	0.95	6	3.50	1.00
15	*bnlg1138*	2.06	0.71	140.88	0.94	5	3.48	1.00
16	*nc 003*	2.06	0.82	257.82	0.98	8	5.54	0.98
17	*umc1108*	2.07	0.66	104.26	0.91	4	2.95	1.00
18	*phi090*	2.08	0.72	198.65	0.96	7	3.55	1.00
19	*umc2077*	2.09	0.61	121.11	0.92	5	2.58	1.00
20	*phi374118*	3.02	0.78	185.54	0.96	6	4.93	1.00
21	*bnlg 1523*	3.03	0.80	254.49	0.98	8	5.49	1.00
22	*umc 2259*	3.03	0.78	185.41	0.96	6	4.61	1.00
23	*phi036*	3.04	0.67	133.37	0.93	5	4.57	1.00
24	*phi 102228*	3.06	0.70	110.11	0.92	4	3.32	1.00
25	*phi 046*	3.08	0.61	145.47	0.94	6	2.59	1.00
26	*bnlg1108*	3.08	0.75	147.45	0.95	5	3.98	1.00
27	*umc1594*	3.09	0.68	133.52	0.94	5	3.09	1.00
28	*phi072*	4.00	0.76	210.52	0.97	7	4.26	0.98
29	*phi096*	4.04	0.78	153.20	0.96	5	4.80	1.00
30	*umc 1175*	4.05	0.60	94.58	0.90	4	2.50	1.00
31	*umc 2284*	4.06	0.86	304.31	0.98	9	7.13	0.96
32	*bnlg252*	4.06	0.76	151.15	0.95	5	4.60	1.00
33	*phi093*	4.08	0.70	137.42	0.94	5	3.14	0.78
34	*nc130*	5.00	0.71	168.19	0.95	6	3.44	0.92
35	*phi024*	5.01	0.80	189.34	0.97	6	5.72	1.00
36	*umc1332*	5.04	0.78	216.81	0.97	7	4.72	0.93
37	*umc2303*	5.05	0.85	334.58	0.98	9	7.73	0.97
38	*phi085*	5.06	0.85	370.80	0.99	9	7.63	0.99
39	*dupssr14*	8.09	0.68	134.47	0.94	5	3.43	0.73
40	*phi 015*	8.08	0.64	126.12	0.93	5	2.62	0.90
41	*umc 1378*	7.00	0.60	118.01	0.92	5	2.66	0.94
42	*bnlg1443*	6.05	0.66	131.04	0.93	5	3.06	1.00
43	*duppsr 28*	4.08	0.70	111.42	0.93	4	3.36	1.00
44	*phi076*	4.11	0.62	98.64	0.91	4	2.66	1.00
45	*phi445613*	6.05	0.69	108.49	0.92	4	2.68	1.00
46	*umc1520*	6.06	0.71	168.34	0.95	6	3.62	1.00
47	*Zag249*	6.01	0.66	131.12	0.93	5	2.94	1.00

PIC = Polymorphic information content, MI = marker index, DI = diversity index, Na = number of actual alleles, Ne = effective alleles, Obs_Hom = observed homozygosity.

**Table 2:** Analysis of molecular variance (AMOVA) among 288 maize inbred lines based on 89 polymorphic SSR markers.

Source	df	SS	MS	Est. Var.	% Var.	F-Stat.	Value	*p*
Between sub-populations	4	931.79	232.95	2.13	7%	F_st_	0.07	0.001
Among individual (within a population)	283	15,781.60	55.77	27.04	88%	F_is_	0.94	0.001
Within individual (across whole population)	288	488.50	1.70	1.70	5%	F_it_	0.95	0.001
Total	575	17,201.89	290.41	30.86	100%			

df: degree of freedom, SS: sum of squares, MS: mean sum of squares, Est. Var.:estimated variance, % Var.: percentage of variation, F_st_—inbreeding coefficient within subpopulations relative to the total, F_is_—inbreeding coefficient within individuals relative to the subpopulation, F_it_—inbreeding coefficient within individuals relative to the total.

**Table 3:** Simple sequence repeats markers found significantly (*p* < 0.001) associated with turcicum leaf blight resistance in marker-trait association analysis done using general linear model.

S. No.	Marker	Bin Location	Physical Position	Tandem Repeats	Marker R^2^	FDR	PIC
1	umc1122 ^#^	1.06	206027905–206027744	(CGT)7	0.17	0.0097	0.76
2	umc2396 ^#^	1.07	NA *	(GTT)5	0.22	0.0003	0.73
3	bnlg1092	2.00	NA *	AG(30)	0.16	0.0200	0.68
4	phi083 ^#^	2.04	42235661–42235792	AGCT	0.13	0.0030	0.71
5	bnlg1138	2.06	NA *	AG(14)	0.22	0.0110	0.71
6	umc1108	2.07	191651695–191651816	(ACGT)4	0.12	0.0012	0.66
7	phi374118	3.02	17628581–17628810	ACC	0.16	0.0110	0.78
8	phi076	4.11	248828113–248828277	AGCGGG	0.11	0.0020	0.62
9	nc130	5.00	1231799–1231940	AGC	0.26	0.0001	0.71
10	phi024	5.01	4540582–4540751	CCT	0.22	0.0200	0.80
11	phi085	5.06	213469971–213469712	AACGC	0.26	0.0023	0.85
12	phi075	6.00	6643571–6643381	CT	0.11	0.0020	0.49
13	umc1520	6.06	169840623–169840474	(GA)8	0.08	0.0164	0.71
14	phi059 ^#^	10.02	8667388–8667241	ACC	0.12	0.0200	0.44
15	umc1367	10.03	26019710–26019551	(CGA)6	0.06	0.0400	0.23

^#^ Markers found significantly associated with TLB resistance in more than one association analysis carried out based on disease response at Bajaura, Srinagar and mean values of both the sites. In this case, the maximum value of R^2^ from either of the association has been mentioned over here. * Marker details are available in the Maize GDB but we could not locate an exact physical position.

## Data Availability

The raw data is available with the first author for any further information/queries. Besides, part of it has been submitted with the manuscript as Tables S1 and S2.

## Supplementary Materials

The following supporting information can be downloaded at: https://www.mdpi.com/article/10.3390/genes13040618/s1, Figure S1: Highly susceptible and resistant maize inbred lines to TLB under artificial inoculated conditions in the field. Figure S2: Neighbor-joining based phylogenetic radial tree showing the genetic relationship among 288 maize genotypes. Three different major clusters each having two sub-groups were found in 288 lines using 89 polymorphic markers. Table S1: Details of 288 genotypes used for genetic characterization and genome-wide association study for TLB resistance. Table S2: Details of 89 SSR markers which were found polymorphic in 288 diverse sets of inbred lines.
