# Supplementary figures and images for: Population Structure Analysis and Association Mapping for Turcicum Leaf Blight Resistance in Tropical Maize Using SSR Markers
Source: Genes (Basel). 2022 Mar 29;13(4):618. doi: 10.3390/genes13040618 (PMC9030036; doi:10.3390/genes13040618)

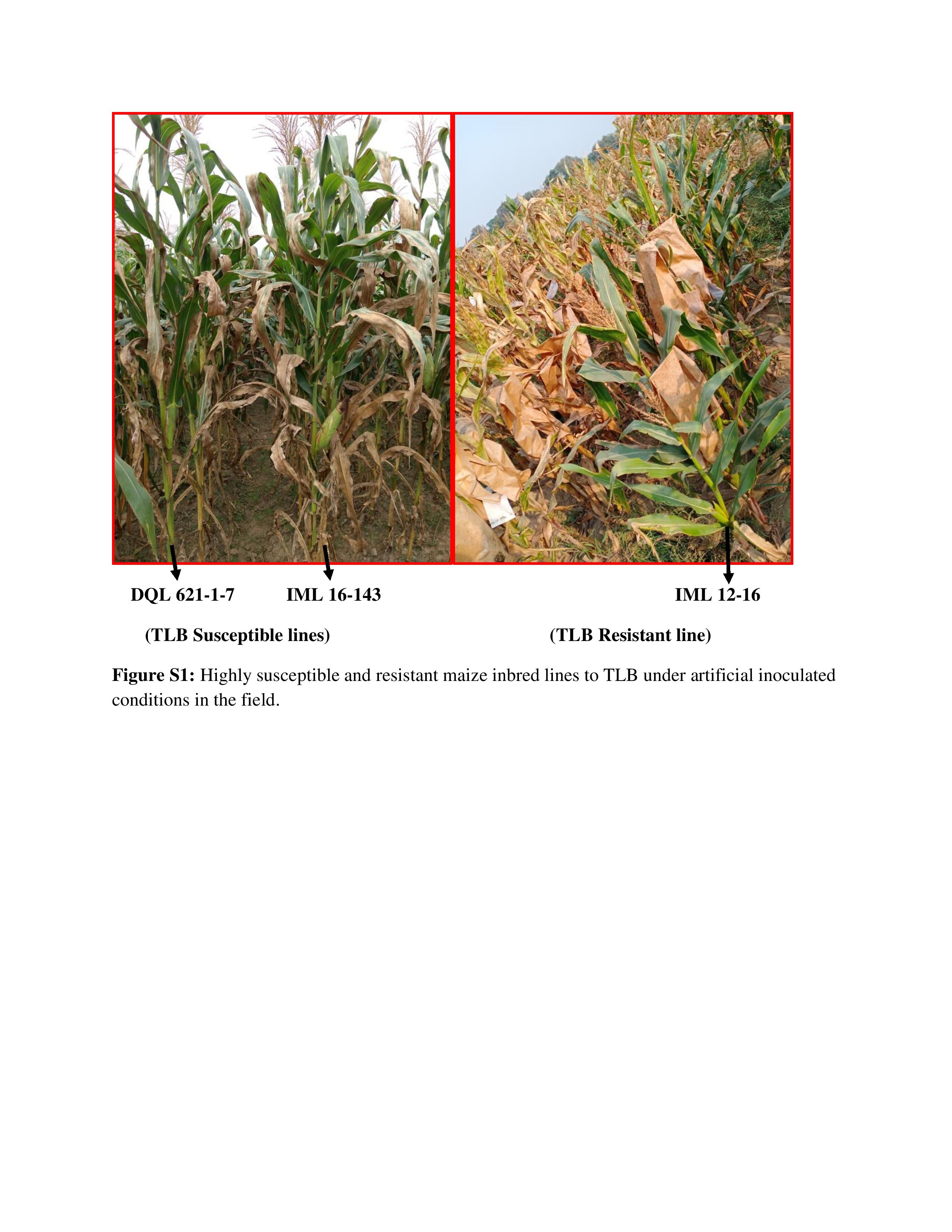

Supplement: Supplementary file 1 [file genes-13-00618-s001.zip › Figure S1.jpg]
